# Using Theory-Based Frameworks to Identify Barriers, Facilitators, Expectations, and Willingness to Pay for Online Medical Consultation Services in China: Qualitative Study

**DOI:** 10.2196/96448

**Published:** 2026-10-06

**Authors:** Shujie Dong, Qiushi Cai, Jingyi Ye, Yanya Chen, Rongsheng Zhao, Wai-kit Ming

**Affiliations:** 1 Department of Pharmacy, Peking University Third Hospital, Beijing, China; 2 Department of Infectious Diseases and Public Health, Jockey Club College of Veterinary Medicine and Life Sciences, City University of Hong Kong, To Yuen Building, 31 To Yuen Street, Hong Kong, China (Hong Kong), 852 34426956; 3 Department of Metabolism, Digestion and Regeneration, School of Medicine, Imperial College London, London, England, United Kingdom; 4 School of Public Health, Li Ka Shing Faculty of Medicine, University of Hong Kong, Hong Kong, China (Hong Kong); 5 School of Nursing, Jinan University, Guangzhou, China

**Keywords:** online medical consultation, public perception, willingness to pay, qualitative research, facilitators, barriers, in-depth interview, AI

## Abstract

**Background:**

Online medical consultation (OMC) services have gained considerable attention as an integral component of telemedicine. Recently, AI has been increasingly integrated into OMC platforms, facilitating more efficient consultations and clinical decision-making. AI-driven OMC services can provide preliminary triage, medication guidance, and diagnostics for multiple medical conditions. Despite the availability and potential benefits of AI-driven OMC services, public acceptance and willingness to pay (WTP) for these services remain low.

**Objective:**

This study aimed to identify perceived barriers, facilitators, expectations, and factors shaping public acceptance of and stated WTP for AI-driven OMC services.

**Methods:**

We conducted semistructured qualitative interviews with patients, caregivers, and health care professionals to explore barriers, facilitators, expectations, and factors shaping public acceptance of and stated WTP for AI-driven OMC services. The study was informed by the theories of perceived risk and perceived benefit, which guided the development of the interview guide. All interviews were audio-recorded and transcribed verbatim. Data were analyzed using NVivo (version 15; Lumivero) with deductive thematic analysis guided by these theories. Coding was conducted independently and cross-checked by 2 researchers to ensure credibility and consistency.

**Results:**

Thematic analysis of 20 in-depth interviews identified 2 main themes and 11 subthemes. Perceived risks and perceived benefits emerged as 2 key perspectives influencing participants’ acceptance and WTP. Psychological, governance, social, functional, health, and financial risks reduced acceptance, whereas convenience, diversity, reliability, efficiency, and educational benefits promoted it. Participants’ self-reported WTP ranged from RMB 0 to RMB 200 (US $0-$27.28; RMB 1=US $0.1364 as of January 15, 2025), with participants who had prior experience with OMC generally reporting higher values than those without prior OMC experience.

**Conclusions:**

This study identified facilitators and barriers influencing public acceptance of and WTP for AI-driven OMC services using theoretical constructs. Our findings offer valuable insights into the development and refinement of AI-driven OMC services, enabling more targeted pricing strategies and tailored services that address public preferences and concerns, as well as supporting the development of standardized regulatory governance for digital medical consultation platforms.

## Introduction

Telemedicine is the delivery of health care services in which distance is a critical factor and health care professionals use information and communication technologies to exchange valid information for the diagnosis, treatment, and prevention of diseases and injuries, as well as for research, evaluation, and education [1]. Online medical consultation (OMC) is an important element of telemedicine and has become increasingly popular over the past few years, especially since the COVID-19 pandemic [2]. Recently, the use of AI in health care has grown rapidly, particularly in the field of telemedicine. Integrating AI into telemedicine has transformed a wide array of medical practices, from diagnostics to medical consultation [3]. The integration of AI into OMC services enables real-time data analysis and decision support, thereby facilitating remote consultations with greater efficiency and accuracy [4]. AI-driven OMC services have been demonstrated to be an effective means of addressing consultations for various medical conditions, such as surgery [5], cancer [6], emergency care [7], psychiatry [8], and cardiovascular diseases [9].

Despite the availability and potential benefits of AI-driven OMC services, their uptake among the public remains low [10]. This gap in adoption highlights the need to understand the multifaceted factors influencing their use [11,12]. Previous studies have predominantly focused on facilitators and barriers associated with OMC services without AI integration. For example, multiple factors have been identified as facilitators of OMC services, including cost savings, reduced waiting and travel times, ease of use, familiarity with the system, trust in technology, family involvement, and patient age [11]. Several factors have also been identified as barriers to OMC services, including slow internet speed, poor audio or video quality, limited internet access, weak signal coverage, difficulty expressing emotions, lack of body language, and user resistance [11]. However, evidence regarding public perceptions of acceptance and willingness to pay (WTP) for AI-driven OMC services remains limited. Although the integration of AI into telemedicine can reshape OMC services, promoting adoption requires addressing both the perceived benefits and risks of these services from users’ perspectives.

The theory of perceived risk suggests that consumers perceive risk due to uncertainty and potentially undesirable consequences associated with paying for or using services [13,14]. It has been widely applied in previous studies to explore public perceptions of health care services, such as mobile health (mHealth) technologies [15], e-consultation services [16], online pharmacy platforms [17], and AI-enabled health care apps [18]. Meanwhile, the theory of perceived benefit emphasizes consumers’ beliefs about the extent to which they will gain positive outcomes from using a specific service [19]. Perceived benefits tend to enhance the intention to use and are associated with health care consumers’ greater willingness to accept the vulnerability (ie, service uncertainty and potential negative consequences) resulting from such uncertainty [13,20]. Understanding perceived risks and benefits is crucial for designing health care services that meet consumer expectations, especially in the context of the rapid development of digital health services and AI technologies. Therefore, the theories of perceived risk and perceived benefit can serve as useful frameworks to evaluate the willingness to accept and pay for AI-driven OMC services.

This study used a qualitative approach informed by the theories of perceived risk and perceived benefit to identify perceived barriers, facilitators, expectations, and factors shaping public acceptance of and stated WTP for AI-driven OMC services. By doing so, it seeks to shed light on the development and refinement of AI-driven OMC services, including targeted pricing strategies and tailored service designs to address patient needs.

## Methods

### Ethical Considerations

This study was approved by the Ethics Committee of Peking University Third Hospital (approval number M20241044). All participants provided informed consent prior to participation. All information collected during the study was treated as strictly confidential. Interview data were anonymized before analysis and stored in NVivo (version 15; Lumivero) on secure, password-protected institutional servers, with access restricted to authorized members of the research team.

### Study Design

In this study, AI-driven OMC services refer to OMC services in which AI technologies are used to support medication-related information provision, preliminary guidance, triage, or decision support. These services may include fully automated AI responses as well as AI-assisted models involving review by health care professionals. As this remains an emerging service model, the study explored participants’ perceptions of this broader service category rather than evaluating a single standardized platform. This study adopted a descriptive qualitative research design to comprehensively explore the factors influencing patients’ acceptance of and WTP for AI-driven OMC services. In-depth semistructured interviews were conducted to collect detailed information from participants.

### Setting and Participants

Purposive sampling was used to recruit participants with diverse characteristics, including sex, age, geographic location, identity characteristics, prior OMC experience, and educational level. The research team approached potential participants from different cities in China through clinical, professional, and personal networks. Some participants also referred other eligible individuals to the research team. Recruitment continued until the sample included patients, caregivers, and health care professionals with varied backgrounds and experiences related to OMC services.

Eligible participants were individuals aged 18 years or older who were able to provide informed consent and participate in a Chinese-language interview. Patients, caregivers, and health care professionals were included to capture diverse perspectives on the factors influencing acceptance of and WTP for AI-driven OMC services. Participants were excluded if they were unable to provide informed consent, unable to complete the interview, or unwilling to be audio-recorded.

In-depth semistructured interviews were conducted in Chinese either face-to-face or remotely via telephone in a quiet and private setting between December 2024 and January 2025. The sample size was determined based on data saturation, with interviews discontinued when no new information emerged [21].

### Data Collection

Before the interviews, participants were informed of the study’s purpose and provided written informed consent. Each interview lasted for 30-45 minutes and was audio-recorded with AI-assisted real-time transcription using a speech-to-text device. All transcripts were checked against the original audio recordings and verified against the recordings within 24 hours to ensure transcription accuracy. All interviews were led by QC, while SD served as the note-taker. Both researchers had backgrounds in pharmacy and qualitative research and were familiarized with the interview guide before data collection. A semistructured interview outline designed based on relevant literature and participant characteristics [22] was pilot tested with 3 volunteers and refined before the formal interviews. The outline consisted of 5 primary questions and was detailed in Textbox 1. Participants were not shown a standardized platform, workflow, use case, or pricing scenario before the interviews. Instead, the interviews used open-ended questions to explore participants’ perceptions, concerns, expectations, and WTP regarding AI-driven OMC services as a broad and emerging service model. Interviews began with open-ended prompts (eg, “what” and “how”) to elicit detailed responses, followed by probing questions for deeper exploration of emerging themes [23,24]. Plain and accessible language was used to encourage open communication. SD documented contextual information to complement the audio-recorded data, including facial expressions and body language in face-to-face interviews and tone, pauses, and emotional reactions in telephone interviews. Participants could withdraw at any time or refuse to answer any question.

Textbox 1. **Semistructured interview outline questions for participants.**

This semistructured interview outline was developed based on the research objectives and core research questions. Relevant interview questions are listed below:

1. Could you briefly share your personal information, including your age, place of residence, family situation, health condition, and internet proficiency?
2. Have you ever used any online medical consultation (OMC) service, particularly one driven by AI technologies? If yes, when would you choose to use such a service? If not, why?
3. How do you feel about AI-driven OMC services? What are their strengths, weaknesses, and key aspects you care about?
4. Would you be willing to use or pay for such services, and what factors might encourage or discourage your willingness to use or pay for them？
5. What are your expectations for AI-driven OMC services, and what areas do you think could be improved or optimized in the future?

### Data Coding and Analysis

The data analysis process was conducted concurrently with data collection, and further sampling decisions and interview questions were guided by the findings of the ongoing data analysis [24]. Interviews were conducted until ongoing coding indicated that no new codes or subthemes relevant to the study objectives emerged [21].

Transcripts were sequentially numbered and imported into NVivo (version 15) for coding. To minimize potential bias and enhance the credibility and consistency of the analysis, 2 researchers independently coded and analyzed the data, followed by cross-checking and discussion to resolve discrepancies. An initial codebook was developed through comparison of independently generated codes and was refined iteratively during the analysis.

The analysis followed Clarke and Braun’s 6-phase thematic analysis approach [25,26]. First, 2 researchers (SD and QC) independently read the transcripts to become familiar with the data. Second, each researcher independently generated initial codes across the dataset, focusing on participants’ perceptions of AI-driven OMC services without applying a fixed coding template. Third, the researchers independently organized related codes into preliminary themes. Initial coding remained open to concepts emerging from the data, while the theories of perceived risk and perceived benefit were used as a sensitizing framework to support theme organization and interpretation. Although the final themes were organized under the broad dimensions of perceived risk and perceived benefit, the subthemes were not predefined by these theories. Instead, they were developed and named by the research team based on recurring patterns in participants’ responses. The 2 sets of codes and preliminary themes were then compared. Differences in coding interpretations, theme boundaries, and theme names were discussed until consensus was reached. Where uncertainty remained, coding and thematic decisions were discussed with the wider research team. Analytic notes were used to document coding decisions, emerging interpretations, and points requiring further discussion. Fourth, the agreed themes were reviewed and refined to ensure internal consistency and clear boundaries. Fifth, the themes and subthemes were refined and named based on the coded data within the broader risk-benefit framework. Finally, the results of the analysis were compiled and presented in a structured report to support the interpretation of WTP and service acceptance.

The final set of codes, themes, and subthemes was jointly reviewed and confirmed by all team members. Representative quotations were selected from the Chinese transcripts to illustrate each theme, translated into English by bilingual researchers, and checked against the original text to preserve participants’ intended meanings.

## Results

### Participant Characteristics

A total of 20 participants were included in this study, comprising 5 male and 15 female participants, aged 22-68 years. Of the 20 interviews, 8 were conducted face-to-face and 12 were conducted remotely via telephone. The sample comprised 15 patients, 3 caregivers, and 2 health care professionals. In terms of geographic distribution, 11 participants lived in urban areas and 9 in rural areas. Regarding educational background, 5 participants held a master’s degree or higher, 9 held a bachelor’s degree, and 6 had completed high school education or below. Detailed participant characteristics are shown in Table 1.

**Table 1.** Characteristics of study participants. An OMC^a^ experience refers to participants’ prior experience with OMC services and does not necessarily indicate prior experience with AI-driven consultation services.

Participant number	Sex	Age (years)	Residence	Identity characteristics	Prior online medical consultation experience	Educational Level
1	Male	41	Urban	Patient	Yes	Master’s degree or above
2	Female	36	Urban	Patient	Yes	Master’s degree or above
3	Male	41	Urban	Caregiver	Yes	Master’s degree or above
4	Female	35	Urban	Patient	Yes	Bachelor’s degree
5	Female	22	Rural	Patient	No	Bachelor’s degree
6	Female	41	Urban	Caregiver	Yes	Bachelor’s degree
7	Female	39	Urban	Patient	Yes	Master’s degree or above
8	Female	34	Urban	Health professional (pharmacist)	No	Bachelor’s degree
9	Female	35	Rural	Patient	No	Bachelor’s degree
10	Female	50	Urban	Patient	No	High school or below
11	Male	24	Rural	Patient	No	Bachelor’s degree
12	Male	30	Rural area	Patient	Yes	Master’s degree or above
13	Female	22	Rural	Patient	No	Bachelor’s degree
14	Female	22	Urban	Patient	Yes	Bachelor’s degree
15	Female	62	Rural	Patient	No	High school or below
16	Male	54	Rural	Health professional (doctor)	Yes	Secondary education, vocational education, or below
17	Female	68	Rural	Patient	No	High school or below
18	Female	25	Urban	Caregiver	Yes	Bachelor’s degree
19	Female	22	Urban	Patient	No	Bachelor’s degree
20	Female	53	Rural	Patient	No	High school or below

a OMC: online medical consultation.

### Thematic Analysis Outcomes

#### Generated Themes: an Overview

Thematic analysis of 20 in-depth interviews identified 2 main themes and 11 subthemes, revealing the multifaceted factors influencing public acceptance and WTP for AI-driven OMC services (Figure 1). Representative participant quotations for themes, subthemes, and WTP are shown in Table 2. These findings reflect the underlying psychological mechanisms and decision-making processes in participants’ evaluations of perceived risks and benefits.

**Figure 1.**
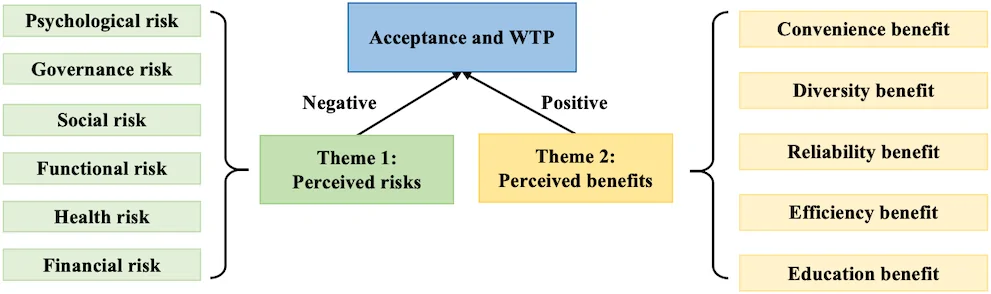
Themes and subthemes identified from thematic analysis of public acceptance and willingness to pay (WTP) of AI-driven online medical consultation (OMC) services, based on perceived risk and perceived benefit theories. WTP: willingness to pay.

**Table 2.** The themes, subthemes, WTP^a^, and participant quotations.

Themes, subthemes, and WTP	Quotations
Theme 1: perceived risks	
Subtheme 1: psychological risk	“I think the process of AI-driven service was complicated for me, so I didn’t use it.” (Participant 17; patient without prior OMC^b^ experience) “I’m not really familiar with it yet, and I haven’t had the chance to try it myself, so I don’t really get how it works. But I guess once I get the hang of it, it might feel a lot easier. I hope the government could promote it more, maybe even have some kind of volunteers or people who can go around teaching how to use it. That would be great, right?” (Participant 20; patient without prior OMC experience) “I don’t really know what AI is. I’m getting on a bit, and I don’t fancy trying out new things like that, so I probably won’t be using it in the future.” (Participant 15; patient without prior OMC experience)
Subtheme 2: governance risk	“Yeah, I mean, although I haven’t used these platforms myself, but I am concerned about the privacy.” (Participant 5; patient without prior OMC experience) “In the future, when AI becomes widely used, how can we ensure that it is not manipulated by certain individuals to create false information? In terms of medical treatment, certain safety assurances need to be provided, particularly in regulatory oversight and control.” (Participant 16; service-participant doctor) “Emm, it’s not an official platform backed by the government right now. So, sometimes I’m not feeling confident about it.” (Participant 3; service-experienced caregiver) “If we’re consulting at a physical place, when there’s a problem, we can quickly identify the person and seek compensation. But for online consultation based on AI, we can’t seek accountability.” (Participant 11; patient without prior OMC experience)
Subtheme 3: social risk	“With AI consultations, we are talking to a robot, not a real person. There’s no face-to-face interaction, which makes it feel less natural. Unlike a doctor..., the AI-driven platform relies only on what you type in, so it might miss important details.” (Participant 12; service-experienced patient) “I feel like, since the AI cannot do a face-to-face consultation, the responses can sometimes be more conservative. This may be because it lacks direct observation and relies solely on the information I provide." (Participant 1; service-experienced patient)
Subtheme 4: functional risk	“Also, since the consulting service provided by AI is just a computer program answering questions, it doesn’t have the authority to prescribe medicine or order tests." (Participant 4; service-experienced patient) “Health care professionals will ask about lots of different things when they’re diagnosing, like your medical history..., AI might not be quite as good at handling these individual details.” (Participant 12; service-experienced patient) “AI lacks extensive clinical experience, and unlike doctors..., AI tends to rigidly follow standard guidelines.” (Participant 18; service-experienced caregiver) “Online consultations aren’t linked to offline medical records, making it hard to review and consolidate information.” (Participant 14; service-experienced patient) “AI still needs training. It might not be well-developed enough to respond like health care professionals.” (Participant 7; service-experienced patient) “Sometimes the system crashed or the internet connection is unstable. It just keeps loading, or the conversation gets wiped out completely. It’s really frustrating when that happens.” (Participant 7; service-experienced patient)
Subtheme 5: financial risk	“What worries me is, if the AI gets it wrong or misses something important, I’ll have to go see a doctor anyway. So I’d be paying twice, first for the AI, then for an actual consultation.” (Participant 3; service-experienced caregiver) “Even if the AI gives some medication advice, I still need a doctor for a prescription. So in the end, I’m paying for both, and that’s just extra hassle and cost.” (Participant 4; service-experienced patient)
Subtheme 6: health risk	“The service should be available the whole day because you don’t know when you might need to ask for advice. In extremely urgent situations, delayed responses can lead to severe outcomes.” (Participant 10; patient without prior OMC experience) “um, sometimes there might even be misunderstandings, you know, there's a risk of things going off track.” (Participant 7; patient without prior OMC experience) “Sometimes, AI doesn’t get it right, ...the information and references aren’t always accurate. If you don’t double-check, you might end up trusting something that’s not actually true.” (Participant 18; service-experienced caregiver)
Theme 2: Perceived Benefits	
Subtheme 7: convenience benefit	“If I can get an appointment, I’d definitely go offline, but often there are no slots available. So, I use online consultations a lot. It’s really accessible.” (Participant 4; service-experienced patient) “AI can handle many users at the same time. People can ask it questions anytime, and it gives us a quick response. It is more flexible” (Participant 11; patient without prior OMC experience) “In rural or remote areas, people may not conveniently go to big cities or top hospitals for consultations. But through this way, they can get medical guidance effectively, and even without having to leave their homes.” (Participant 16; doctor without prior OMC experience) “...But for some small issues that I’m unsure about or I have a general understanding of my condition but encounter further questions or uncertainties later, I think these can easily be addressed through online consultations conducted by AI.” (Participant 1; service-experienced patient)
Subtheme 8: diversity benefit	“Personally, I’m not very fond of oral communication. For text-based consultations, it’s easier for me to explain things. I prefer offline interactions with doctors to avoid any potential awkwardness.” (Participant 12; service-experienced patient) “A nice thing about online consultation is I can choose different consultation ways according to my needs. And there’s no limit on how many messages you can send.” (Participant 2; service-experienced patient) “For example, if I feel that I have a mental illness and choose to take medication, I will go to the Internet for consultation, because I do not want to let others know.” (Participant 9; patient without prior OMC experience)
Subtheme 9: reliability benefit	“Theoretically speaking, if it’s powerful enough, I think it might integrate information from different sources and give more comprehensive advice. In an ideal scenario, it might even be more professional because its memory and storage capacity could be stronger compared to humans. Also, it can retrieve information faster in some ways, so yeah, that’s what I think.” (Participant 18; service-experienced caregiver) “If AI can supplement the answers from health care professionals it would be more comprehensive and reliable.” (Participant 1; service-experienced patient) “I’d trust AI more if a doctor reviewed its recommendations before they were given to patients, ensuring accuracy and safety.” (Participant 19; patient without prior OMC experience)
Subtheme 10: efficiency benefit	“AI consultations save time because I can quickly get basic medical information without waiting for a doctor to explain everything. Plus, it’s free, so there’s no cost at all.” (Participant 6; service-experienced caregiver) “Sometimes, I don’t have time to visit a doctor in person, so using AI is much more convenient. It’s free or at least much cheaper than online consultations with real doctors.” (Participant 2; service-experienced patient) "I can describe my symptoms to AI, and it helps me figure out which department I should visit. That way, I don’t waste time wondering where to go when I actually need to see a doctor.” (Participant 14; service-experienced patient)
Subtheme 11: education benefit	“If patients first use AI to get some initial information about their medication or health concerns, they can communicate more clearly and confidently with pharmacists. This often helps us give them more accurate and effective treatment.” (P8; pharmacist without prior OMC experience) “Health care professionals might not always be familiar with every medication, especially if it’s outside their specialty area. In such cases, AI can really help by providing more detailed information.” (P16; service-experienced doctor) “I can first check with the AI, get some background knowledge, and this helps me discuss things better with the doctor later. Also, by using AI, I get to learn more about medication use, drug interactions, and overall health education.” (P14; service-experienced patient)
WTP	“To be honest, I just believe AI consultations should be free. It’s not like there’s an actual doctor involved. It’s just pulling stuff from the internet, so why should we pay for that?” (Participant 11; patient without prior OMC experience) “If it’s just an AI spitting out answers, I wouldn’t pay for it. But if there’s a proper doctor checking things and making sure the advice is right, then yeah, that’s different. That’s worth paying for. I’d be willing to pay up to US $30 for that.” (Participant 12; service-experienced patient) “Maybe they could do it in levels—basic AI stuff for free, but if you want a real person to review it, then you pay a bit. At least that way, people can decide what they need.” (Participant 18; service-experienced caregiver)

a WTP: willingness to pay.

b OMC: online medical consultation.

#### Theme 1: Perceived Risks

##### Subtheme 1 (Psychological Risk): Unfamiliarity and Skepticism Toward the Services

Many participants without prior OMC experience were skeptical about the reliability of online and AI-driven services and tended to prefer in-person interactions with doctors or pharmacists. Some expressed unfamiliarity with AI-driven medical consultation services, leading to apprehension and reluctance to use the platform. One participant had never heard of AI being used for medical consultations and showed little interest in adopting such technology. Additionally, several participants without prior OMC experience found the AI system difficult to navigate and often required assistance from others to access the service. Their hesitation was further reinforced by a lack of prior experience with similar digital health care platforms. Without adequate information, they found it difficult to recognize the potential benefits of AI-driven consultations. Training provided by health care professionals through video tutorials or user manuals could help them feel more confident in using the service.

##### Subtheme 2 (Governance Risk): Data Privacy, Security, and Institutional Accountability

Quite a few participants voiced worries over inadequate governance of AI-driven OMC platforms, covering 2 core dimensions, namely data privacy and protection and institutional accountability. Many feared that their confidential medical and personal data might be disclosed to unauthorized third parties, which would erode their trust in digital consultation services. More importantly, participants raised prominent governance loopholes regarding regulatory supervision and liability clarification. Some noted that current AI consultation platforms are not operated by nationally certified authorities, making it unclear where to file complaints or seek redress in cases of inaccurate advice or service failures. The lack of defined responsibility and regulatory oversight left them hesitant to rely on AI-driven consultations. Many participants indicated that they would only consider using and paying for such services if robust data protection measures and transparent mechanisms for complaint handling and accountability were established. Some noted that current AI consultation platforms are not operated by nationally certified authorities, making it unclear where to file complaints or seek redress in cases of inaccurate advice or service failures. The lack of defined responsibility and regulatory oversight left them hesitant to rely on AI-driven consultations. Many participants indicated that they would only consider using and paying for such services if robust data protection measures and transparent mechanisms for complaint handling and accountability were established.

##### Subtheme 3 (Social Risk): Limited Interpersonal Interaction and Communication Quality

Participants pointed out that one of the shortcomings of AI-driven OMC services was the lack of face-to-face interaction. Without body language or eye contact, they felt uneasy and found it difficult to convey complex information. Some also criticized the overly cautious nature of AI responses, which often suggested possibilities such as “might have” or “could be” instead of giving clear conclusions, leaving users uncertain. The quality of advice also depended on the completeness of the information provided. When users lacked medical knowledge, struggled to describe symptoms, or had no test results, responses became vague and less useful.

##### Subtheme 4 (Functional Risk): Limited Clinical, Technical, and Usability Functionality of Platforms

Service-experienced participants indicated that current AI-driven consultation platforms still have functional limitations. Although these systems can analyze user inputs, provide basic drug information, and suggest possible treatment options, they cannot prescribe medications, request laboratory tests, or perform full medical evaluations. Patients must still consult health care professionals for accurate diagnoses, prescriptions, and examinations to ensure safe and effective care. Many participants also noted that AI performs poorly in handling highly individualized medical problems because it lacks clinical experience. It cannot independently access biomarkers or imaging data and depends entirely on user-provided information, which restricts its capacity to offer personalized advice. In addition to these clinical limitations, participants also raised usability-related concerns, including poor network connections, system errors, software glitches, and difficulty navigating the platform. These issues reduced their confidence in the stability and reliability of AI-driven OMC services. As these technologies are still at an early stage, some participants expressed concerns about technical stability, incomplete data integration, and limited accuracy due to insufficient real-world validation.

##### Subtheme 5 (Financial Risk): Uncertain Pricing and Potential Additional Health Care Costs

Participants expressed concerns that if AI-generated recommendations were inaccurate or incomplete, they might spend more on unnecessary treatments or follow-up consultations with health care professionals. Additionally, since AI lacks the authority to prescribe medications, users still need to consult doctors for prescriptions and laboratory tests, incurring further costs. These financial uncertainties made participants hesitant to invest in AI-driven consultation services, highlighting the need for clear pricing structures and transparent value propositions to justify any associated fees.

##### Subtheme 6 (Health Risk): Potential Health Harm From Delayed or Inaccurate Advice

Several participants mentioned that some AI-driven consultation services were embedded in company-developed platforms operating within fixed service hours. Although AI was always technically available, responses were sometimes constrained by platform schedules, leading to concerns about the ability to address urgent medical problems during the night. Some participants felt that delayed professional feedback, due to restricted hours or limited consultant availability, might increase health risks. Others expressed worries about the accuracy of AI-generated recommendations. Service-experienced users noted that communication during online consultations was less smooth than in face-to-face settings, which sometimes led to misunderstandings between patients and health care professionals. A few participants further reported that AI occasionally produced incorrect information or cited nonexistent references, creating a false sense of reliability.

### Theme 2: Perceived Benefits

#### Subtheme 1 (Convenience Benefit): Accessible and Flexible Services

Participants highlighted accessibility as a major factor promoting the use of AI-driven OMC services. Those living in rural or underserved areas described these platforms as a convenient alternative for obtaining medical information and guidance, helping them overcome geographical barriers. Participants from larger cities also recognized the convenience of AI-driven consultations compared with traditional health care visits. Many appreciated the flexibility to seek medical advice at times that suited their schedules, often from home or other comfortable locations. Several participants mentioned that AI-driven consultations were useful for addressing minor health issues or follow-up questions after outpatient visits.

#### Subtheme 2 (Diversity Benefit): Multiple Consultation Formats and Language Options

By offering diverse communication methods and language options, AI-driven online consultation services foster a more inclusive health care experience. Participants indicated that they could choose between voice, text, or video formats according to their preferences. These communication options were also considered advantageous for discussing sensitive health topics, such as mental health, as they offered greater privacy and reduced the discomfort associated with face-to-face communication. In particular, some participants who were more introverted preferred text-based consultations because they found them less stressful than speaking directly with health care providers. Additionally, multilingual support further enhanced inclusivity, enabling users from different linguistic or dialect-speaking backgrounds to seek medical advice without language barriers.

#### Subtheme 3 (Reliability Benefit): Comprehensive Medical Information via Data Integration

Most participants regarded AI-driven medical consultation services as relatively reliable due to their ability to process large volumes of data and integrate multiple information sources. Unlike human consultations, these systems can rapidly analyze clinical guidelines, drug information, and statistical data to provide evidence-based recommendations. Several participants emphasized that AI’s capacity to efficiently retrieve and cross-reference information could enable more comprehensive advice. Furthermore, several participants suggested that integrating AI-generated insights with human consultations could provide more comprehensive and trustworthy guidance, as it supplements health care professionals’ responses with additional information. Others believed that implementing a system in which AI-generated recommendations undergo human review before being delivered to patients would further enhance reliability, accuracy, and patient safety.

#### Subtheme 4 (Efficiency Benefit): Reduced Health Care Costs and Time

Most participants highlighted the cost-effectiveness and time-saving advantages of AI-driven medical consultation services. Unlike traditional online consultations, which can be expensive, AI services are either free or substantially more affordable, making them a cost-efficient option. They also help save time by providing quick access to basic medical information, reducing the need for lengthy explanations from health care professionals. Additionally, some participants noted that AI can assist in identifying potential health issues and recommending appropriate specialist departments, thereby streamlining the process before in-person visits.

#### Subtheme 5 (Education Benefit): Improved Health and Medication Literacy

Most participants emphasized that AI-driven medical consultation services play an important role in improving patient education and medical literacy. Participants found that AI’s ability to clearly explain medication indications, dosage instructions, mechanisms of action, potential drug interactions, and relevant medical concepts helped them better understand their treatment plans. This enhanced their readiness for face-to-face consultations and facilitated communication with health care providers. AI-driven consultations were perceived as effective tools for empowering patients, promoting informed decision-making, and increasing confidence in managing their medications and health conditions.

### WTP

The majority of participants assumed that AI-driven services should be free. Some believed that AI-driven online consultation services could be priced according to their level of human involvement. For example, fully automated AI-driven services that rely solely on algorithmic responses were expected to remain free or low-cost, as they primarily aggregate publicly available medical information. In contrast, AI-assisted services involving health care professionals could be charged, as expert validation and personalized advice make a paid model more justifiable. A tiered pricing model could balance accessibility and quality, offering free basic AI consultations while charging for expert-reviewed recommendations. Participants who preferred traditional offline consultations reported lower WTP, whereas those satisfied with prior OMC experiences showed higher WTP for such services. In summary, participants reported WTP values ranging from RMB 0 to RMB 200 (US $0-$27.28; RMB 1=US $0.1364 as of January 15, 2025), with some indicating a higher WTP for services reviewed or validated by senior professionals or tertiary hospitals.

## Discussion

### Principal Findings

This qualitative study explored perceived barriers, facilitators, expectations, and factors shaping public acceptance of and stated WTP for AI-driven OMC services. Through in-depth interviews with 20 participants from diverse backgrounds, perceived risks and perceived benefits were identified as 2 key dimensions influencing participants’ acceptance and WTP for these services. Regarding perceived risks, psychological, governance, social, functional, health, and financial risks were found to negatively affect acceptance, serving as potential barriers that may discourage engagement with AI-driven OMC services. For perceived benefits, convenience, diversity, reliability, efficiency, and educational benefits emerged as key motivating factors encouraging acceptance. Participants reported WTP values ranging from RMB 0 to RMB 200 (US $0-$27.28; RMB 1=US $0.1364 as of January 15, 2025), with participants’ views appearing to vary by their prior OMC experience and understanding of the service model. These findings provide valuable insights for the development and optimization of AI-driven OMC services, supporting targeted pricing strategies and personalized service design that address public preferences and concerns.

The theory of perceived risk provides a valuable framework for interpreting participants’ hesitancy toward AI-driven OMC services [27]. As novel health care technologies often face initial resistance due to unfamiliarity [28], psychological risk was reflected in participants’ lack of familiarity with AI-driven platforms, leading to skepticism about reliability and a preference for face-to-face interactions. The preference for in-person consultations reflects not only technological hesitancy but also established expectations regarding health care delivery models [29]. Governance risks emerged as major concerns, with participants expressing fears of data leakage and inadequate regulatory oversight. These findings align with previous studies that highlighted concerns about ethical dilemmas, unclear regulations, and data privacy in health care–related AI applications [30,31]. Lack of transparency remains a prevalent issue across most existing AI technologies [32]. Social risk was evident in participants’ discomfort with non–face-to-face communication, which they felt hindered the expression of complex health information. Deficiencies in empathetic responses and human-centered care make AI interactions less acceptable to some users [33]. Functional risks were associated with both clinical and technical limitations of the platforms, including the inability to prescribe medications, conduct tests, or deliver highly personalized advice, which reduced the perceived value of the service. Beyond clinical functionality, usability appears to be an important factor shaping trust and acceptance, as technical stability, ease of navigation, and platform reliability influence whether users feel confident relying on AI-driven OMC services. AI-driven consultations were considered more reliable for managing straightforward tasks and therefore more acceptable [34], although technical failures may compromise reliability and erode trust among health care professionals [32,35]. Health risks centered on urgent care availability and potential misinformation, while financial risks reflected uncertainty about whether AI-driven services would ultimately reduce or increase health care costs. These multifaceted risks collectively present substantial barriers to the adoption of and WTP for AI-driven OMC services, particularly among participants without prior OMC experience.

Perceived benefits emerged as key motivating factors for WTP for AI-driven OMC services. Importantly, these benefits refer to participants’ perceived benefits rather than demonstrated performance of the AI-driven OMC model, as this study did not evaluate a standardized platform or clinically validate the accuracy, safety, or reliability of medication advice. OMC may extend health care to marginalized populations through remote consultations, monitoring, and technology-assisted diagnosis [36], and AI is expected to provide more real-time predictions of patient outcomes in the future [37]. AI has expanded access to integrated health care by enhancing provider communication, facilitating interactions, and serving as a knowledge hub [38,39]. Such flexibility aligns with increasing consumer demand for on-demand health care [40,41]. Service diversity, including multiple communication modes and language support, created a more inclusive health care experience appealing to diverse user preferences and communication styles. Reliability was attributed to AI’s ability to process and integrate large datasets [42], although hybrid models with human oversight were considered more trustworthy. While user-friendly AI platforms are essential for implementation, integrating them into existing health care systems remains challenging and often requires substantial changes to established operational workflows [43]. Potential cost-effectiveness was perceived as another benefit, with participants expecting time or cost savings compared with traditional consultations. Moreover, participants perceived educational value in these services, particularly for improving medication knowledge, preparing for subsequent consultations, and supporting communication with health care professionals. However, whether these perceived benefits translate into improved clinical outcomes requires further evaluation.

Regarding WTP, our findings reveal that participants’ WTP was shaped by how they understood the service model and the level of human involvement. Many participants believed that fully automated AI-driven OMC services should be free or low-cost, whereas services involving review by health care professionals were considered more acceptable as paid services. These findings align with the broader digital health literature showing that WTP is highly context-dependent and varies by service modality, user characteristics, and the perceived value of the service. A systematic review of eHealth WTP studies identified substantial heterogeneity across studies: one-time WTP ranging from US $0.88 to US $191.84 and monthly WTP ranging from US $5.25 to US $45.64, with WTP differing by population characteristics and delivery modality [44]. Another study of primary care web portals similarly suggested that patients may be willing to pay modest fees for online services that offer direct practical value, such as physician messaging, medical record access, and medication refills [45]. More recent evidence from health apps in Hong Kong also showed that WTP was associated with prior app experience and perceived benefit, while unwillingness to pay was commonly linked to expectations that such services should be free, distrust, and limited awareness of benefits [46]. In line with previous studies, our participants appeared to attach value less to AI involvement itself than to clinically meaningful service attributes, particularly professional oversight, trustworthiness, convenience, and affordability. Since WTP was elicited through open-ended qualitative questions and participants discussed different service models, including AI-only and professionally reviewed services, this range should be interpreted as exploratory evidence of perceived value rather than as a pricing estimate. As highlighted by a previous study on cost-aware frameworks, health care AI development expenses—including data acquisition, computational resources, and developer labor—are frequently underestimated in conventional cost assessments [47]. Rather than supporting a specific payment model, our findings suggest that future payment models for AI-driven OMC services need to balance affordability, perceived value, professional oversight, and equitable access. The strategic design of AI payment models is therefore crucial to enhance WTP while optimizing financial efficiency and ensuring equitable access across populations [48]. In the future, insurance companies or health networks may leverage reimbursement policies and out-of-pocket costs to influence or restrict patients’ provider choices as part of cost-effectiveness–based payment strategies [49]. Prior OMC experience may have shaped how some participants described service value and payment conditions; however, this study did not formally compare WTP across experience groups. The experience-related observations, which refer to prior OMC experience with or without the involvement of AI, should be interpreted as exploratory and hypothesis-generating rather than as evidence of subgroup differences in WTP. Some participants with prior OMC experience provided more concrete descriptions of payment conditions, whereas participants without such experience more often expressed uncertainty or expected AI-only services to be free. Variations in preferred service models may also reflect personal characteristics, prior digital health experiences, perceived needs, and expectations [50]. These findings suggest that trial periods or freemium models may be worth further exploration to build initial trust, although future quantitative or experimental studies using standardized service descriptions and payment scenarios are needed before drawing conclusions about pricing models.

### Implications

By identifying participants’ perceived barriers, facilitators, expectations, and payment considerations, our findings provide user-centered implications for the design, implementation, and future evaluation of AI-driven OMC services. This study used a qualitative approach, informed by the theories of perceived risk and perceived benefit, to identify perceived barriers, facilitators, expectations, and factors shaping public acceptance of and stated WTP for AI-driven OMC services. These findings suggest that service design may need to consider not only technical performance but also usability, trust, privacy protection, professional oversight, and affordability from the user perspective. For policymakers, establishing clear regulatory frameworks for AI-driven OMC services could help address privacy concerns and build public trust. Regulations should balance innovation with consumer protection. For telemedicine developers, our results highlight the importance of intuitive, user-friendly interfaces to overcome psychological barriers associated with unfamiliarity. Integrating AI-generated recommendations with human oversight could mitigate concerns about accuracy, personalization, and accountability. From a payment perspective, tiered service models that distinguish between basic AI-only support and professionally reviewed services may be worth further exploration, although future quantitative studies are needed to evaluate WTP and pricing models more precisely.

### Limitations

Several limitations should be considered when interpreting our findings. First, the sample size of 20 participants, while appropriate for qualitative research, limits the transferability of the results to broader populations and settings. Although patients, caregivers, and health care professionals were included to capture different stakeholder perspectives, the small numbers of caregivers and health care professionals limited the ability to compare views across groups or determine whether saturation was achieved within each stakeholder group. Therefore, subgroup-related findings should be interpreted as exploratory rather than representative of each stakeholder group.

Second, the study was conducted during a period when AI-driven OMC services were in the early stages of adoption in China, suggesting that perceptions may evolve as these services become more mainstream. Third, the focus on stated rather than revealed WTP may not fully capture actual payment behaviors in real-world settings. In addition, WTP was explored through open-ended qualitative questions rather than a formal contingent valuation approach, and participants were not asked to evaluate one standardized service model or payment scenario. Therefore, the reported WTP values should be interpreted as exploratory observations of perceived value rather than pricing estimates. Participants’ descriptions of AI as efficient, comprehensive, or reliable reflected perceived or expected attributes rather than empirically tested properties of a specific AI-driven OMC platform. Future studies should evaluate the clinical accuracy, safety, and reliability of AI-generated medical advice under standardized service scenarios. Fourth, because the interviews were conducted in Chinese and selected quotations were translated into English for reporting, some linguistic nuances may have been affected during translation, although translated quotations were checked against the original transcripts to preserve participants’ intended meanings. Although some findings may be relevant to countries with similar health care contexts, such as uneven medical resource distribution, growing telemedicine use, and early-stage AI integration, transferability should be considered cautiously. Health care financing, regulation, pharmacist roles, and public trust in AI may differ across settings. Future research should address these limitations through larger quantitative studies, cross-cultural comparisons, and experimental designs measuring actual payment behaviors rather than stated intentions.

### Conclusion

Through semistructured in-depth interviews with 20 participants from diverse backgrounds, perceived risks (psychological, governance, social, functional, health, and financial risks) and perceived benefits (convenience, diversity, reliability, efficiency, and educational benefits) were identified as 2 key dimensions associated with participants’ acceptance and WTP for AI-driven OMC services. These findings provide exploratory insights into participants’ concerns, perceived value, and expectations, which may inform future service design and further research on payment models for AI-driven OMC services.

## Supplementary material

10.2196/96448 Checklist 1 COREQ checklist.

## Abbreviations

mHealth: mobile health
OMC: online medical consultation
WTP: willingness to pay
